# Diagnostic accuracy of PCR for detecting ALK gene rearrangement in NSCLC patients: A systematic review and meta-analysis

**DOI:** 10.18632/oncotarget.17914

**Published:** 2017-05-17

**Authors:** Xia Zhang, Jian-Guo Zhou, Hua-Lian Wu, Hu Ma, Zhi-Xia Jiang

**Affiliations:** ^1^ Department of Nursing, Affiliated Hospital of Zunyi Medical College, Zunyi 563000, China; ^2^ Department of Oncology, Affiliated Hospital of Zunyi Medical College, Zunyi 563000, China

**Keywords:** polymerase chain reaction, anaplastic lymphoma kinase, carcinoma, non-small cell lung, systematic review

## Abstract

**Background:**

Anaplastic lymphoma kinase (ALK) gene fusion has been reported in 3∼5% non-small cell lung carcinoma (NSCLC) patients, and polymerase chain reaction (PCR) is commonly used to detecting the gene status, but the diagnostic capacity of it is still controversial. A systematic review and meta-analysis was conducted to clarify the diagnostic accuracy of PCR for detecting ALK gene rearrangement in NSCLC patients.

**Results:**

18 articles were enrolled, which included 21 studies, involving 2800 samples from NSCLC patients. The overall pooled parameters were calculated: sensitivity was 92.4% [95% confidence interval (CI): 82.2%–97.0%], specificity was 97.8% [95% CI: 95.1%–99.0%], PLR was 41.51 [95% CI: 18.10–95.22], NLR was 0.08 [95% CI: 0.03–0.19], DOR was 535.72 [95% CI: 128.48–2233.79], AUROC was 0.99 [95% CI: 0.98–1.00].

**Materials and Methods:**

Relevant articles were searched from PubMed, EMBASE, Web of Science, Cochrane library, American Society of Clinical Oncology (ASCO), European Society for Medical Oncology (ESMO), China National Knowledge Infrastructure (CNKI), China Wan Fang databases and Chinese biomedical literature database (CBM). Diagnostic capacity of PCR test was assessed by the pooled sensitivity and specificity, positive likelihood ratio (PLR), negative likelihood ratio (NLR), diagnostic odds ratio (DOR), area under the summary receiver operating characteristic (AUROC).

**Conclusions:**

Based on the results from this review, PCR has good diagnostic performance for detecting the ALK gene fusion in NSCLC patients. Moreover, due to the poor methodology quality of the enrolled trials, more well-designed multi-center trials should be performed.

## INTRODUCTION

Lung cancer is the leading cause of cancer-related death globally. In 2013, tracheal, bronchus and lung (TBL) cancer has led 1.6 million deaths for 188 countries [1]. In 2011, the death of lung cancer accounts for 27.08% in men and 21.47% in women of the ten highest mortalities cancers in China [2]. Non-small cell lung carcinoma (NSCLC) accounts for approximately 85% of lung cancer and its prognosis remains very poor [3, 4]. Anaplastic lymphoma kinase (ALK) encodes a receptor tyrosine kinase which is normally expressed only in select neuronal cell types. However, with the fusion gene that joints the echinoderm microtubule-associated protein-like 4 (EML4) gene with ALK gene was found in a subset of NSCLC in 2007 [5] and other fusion genes with ALK have been reported in NSCLC Subsequently [6, 7], ALK translocation has been considered to be a potential oncogenic driver and an important therapeutic target in NSCLC. Chiari R et al also reported that NSCLC patients harboring ALK gene translocation could be benefit from tyrosine kinase inhibitor treatment [8, 9]. Therefore, it’s vital to identify the ALK gene status to implement targeted therapy in NSCLC patients with ALK fusions.

Currently, the common detection methods for ALK translocation are fluorescent *in situ* hybridization (FISH), polymerase chain reaction (PCR), immunohistochemistry (IHC), and FISH is regarded as the gold standard to identify the ALK-positive patients for crizotinib therapy. Up to now, reverse transcription polymerase chain reaction (RT-PCR) has been used to identify ALK rearrangement in many studies which showed that RT-PCR was a highly sensitive and specific technique that made it possible for detection even a few molecules of chimeric ALK transcripts [10–13]. Additionally, the multiplex RT-PCR can sensitively detect not only certain ALK fusion gene variant even in formalin-fixed paraffin-embedded (FFPE) tissue sections, but also the abundance of EML4-ALK positive cells in NSCLC tumor tissues [14–16]. However, given the unknown variants of ALK alters and mRNA degradation in FFPE tissues, the sensitivity of PCR remains controversial [17–19]. What’s more, systematic review and meta-analysis related to evaluating the diagnostic accuracy of PCR has not been reported up to now. Therefore, we regarded FISH as the reference standard, PCR as the index test, undertook a systematic review and meta-analysis to evaluate the diagnostic values of them for identifying ALK gene rearrangement in patients with NSCLC. We have followed the PRISMA chart displayed in Supplementary document 1 (see Suppplementary Document 1 in supplementary material available online at HYPERLINK “http://www.prisma-statement.org/PRISMAStatement/PRISMAStatement.aspx). Additionally, The protocol is registered with the Centra for Reviews and Dissemination PROSPERO database (Available at: https://www.crd.york.ac.uk/PROSPERO/display_record.asp?ID=CRD42015019905, Registration No.CRD42015019905)

## RESULTS

### Study characteristics

**Figure 1:**
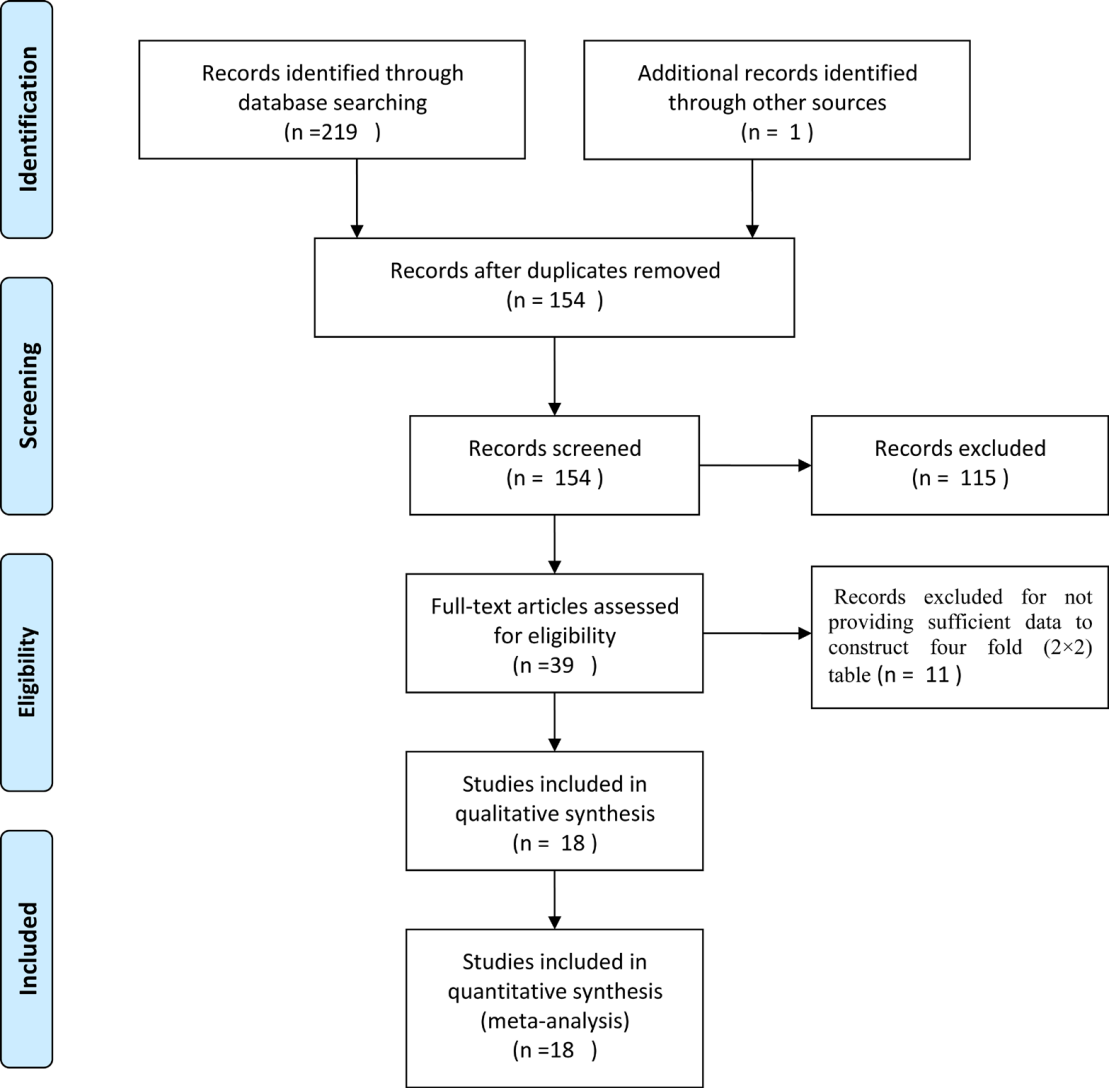
Flowchart for identification of studies

Figure 1 shows the results of the literature searched and selected which followed the PRISMA (Supplementary Document 2). A total of 220 studies were identified by primary literature search and manual review of reference lists. 66 studies were excluded as duplicates. After reviewing the titles, abstracts, 115 articles were excluded and 39 potentially included were reading for full-text. Finally, 18 studies [10, 13, 17–19, 20–32] were enrolled in this systematic review and meta-analysis, including 405 reference-positive samples and 2395 reference-negative samples from NSCLC patients. Studies were from China, Czech Republic, America, Japan, France and Finland between 2012 and 2015. In the studies included, the types of tumor were NSCLC, lung adenocarcinomas; types of tissue were tumor tissue and pleural effusion; ALK fusion were EML4-ALK and ALK; specimens for PCR and FISH were FFPE, cytological smears and fresh frozen; the types of PCR were quantitative real time reverse transcription polymerase chain reaction (qRT-PCR) and end-point PCR, the cut-offs for FISH in the included studies were different, and most studies hadn’t reported the principle of PCR. Detailed information of main characteristics for the included studies was shown in Supplementary Table 1.

### Method quality

**Figure 2:**
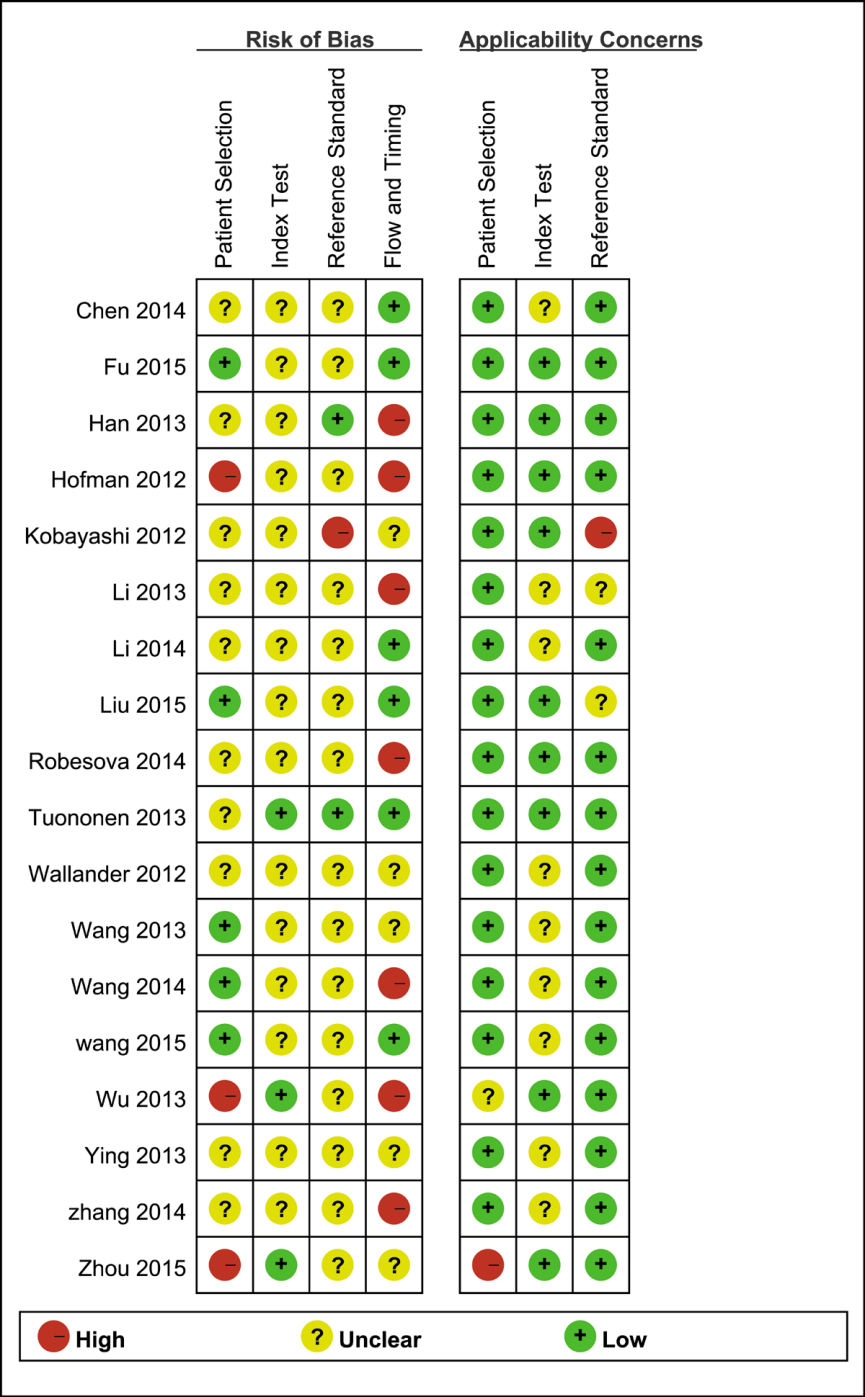
Risk of bias and applicability concerns summary review authors’ judgements about each domain for each included study

All the eligible studies have received methodological quality assessment with the QUADAS-2 tool and results are listed in Figure 2 and Supplementary Figure 1.

### Diagnostic performance

**Figure 3:**
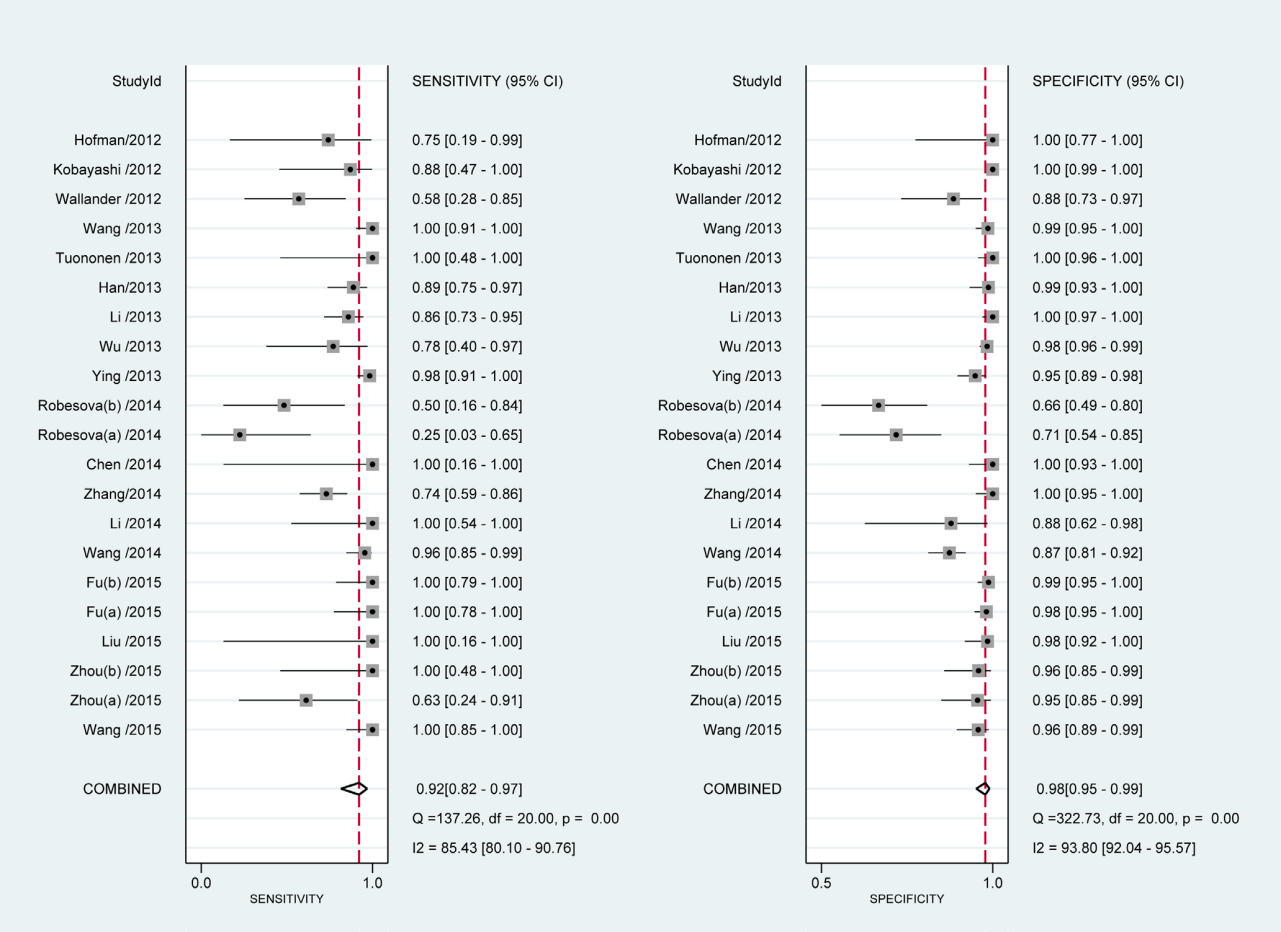
Meta-analysis of sensitivity and specificity of PCR

**Figure 4:**
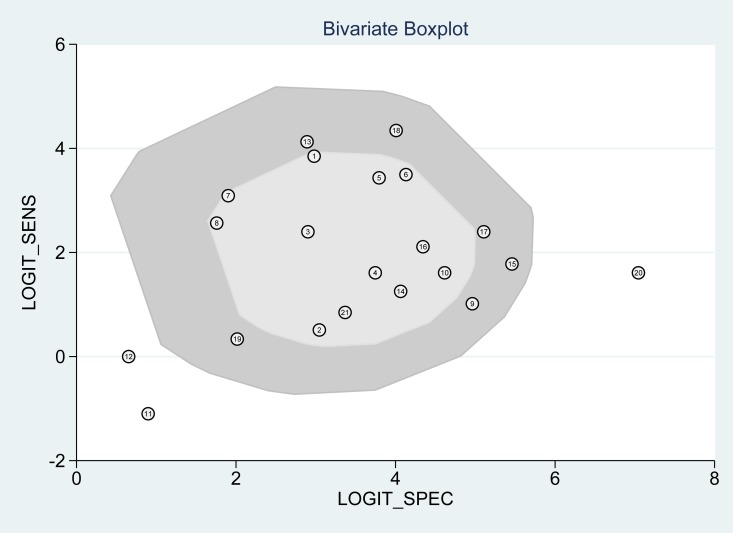
Bivariate boxplot of the studies included

**Figure 5:**
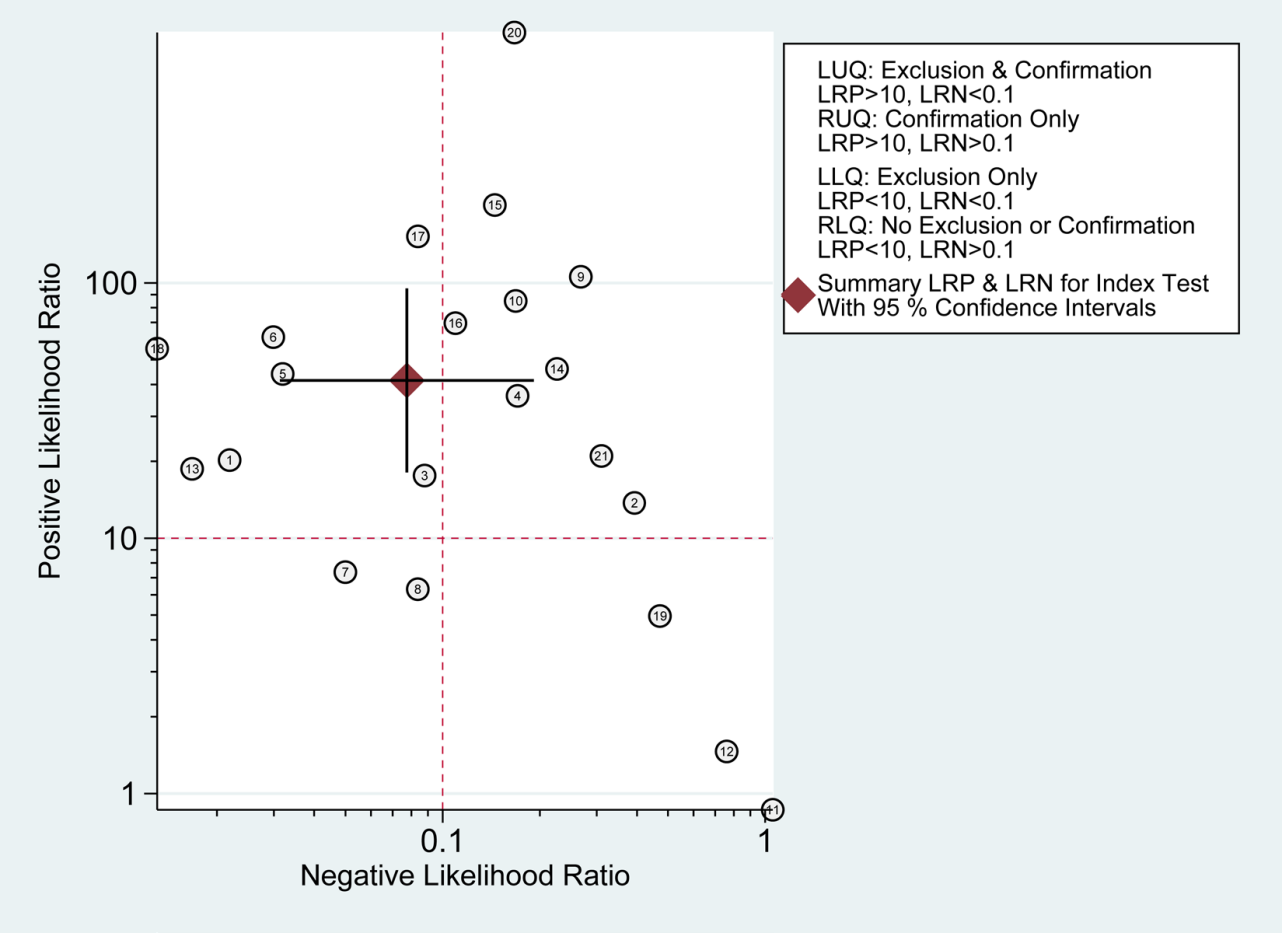
Likelihood ratio scattergram of PCR

**Figure 6:**
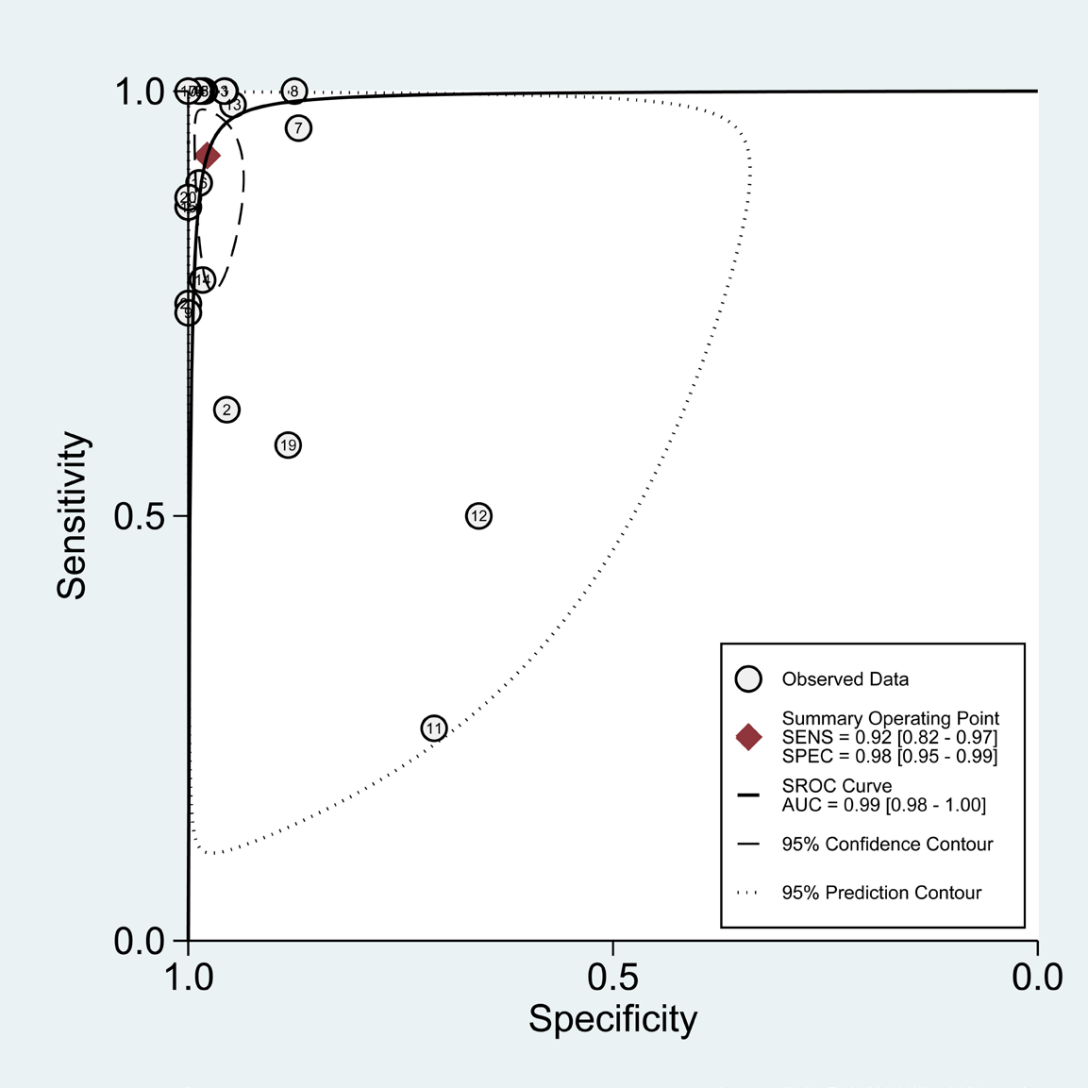
The SROC curve of the PCR assay for detecting the ALK rearrangement

**Figure 7:**
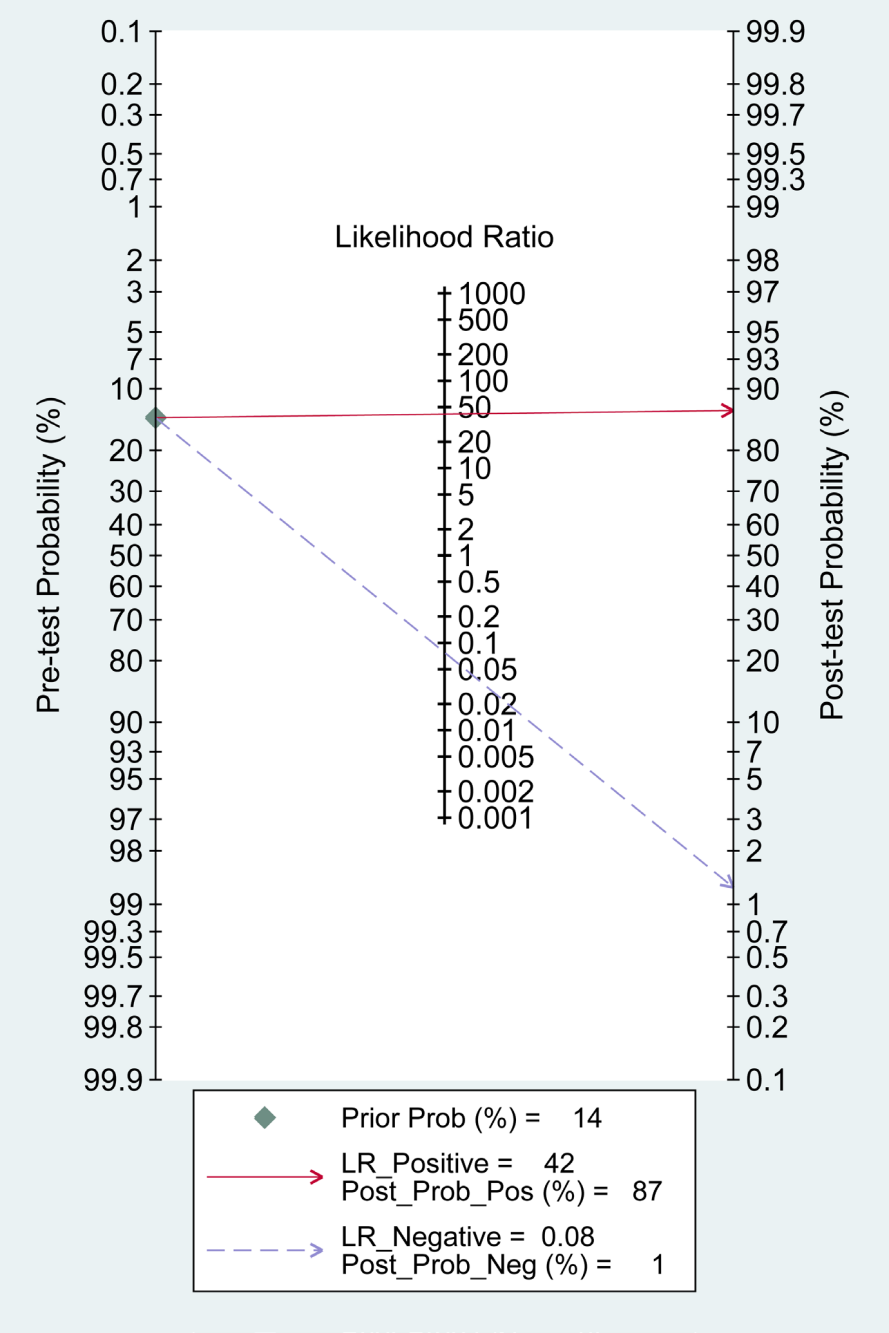
Fagan diagram assessing the overall diagnostic value of PCR for detecting ALK rearrangement

According to the bivariate regression model, the overall pooled sensitivity was 0.92 (0.82–0.97) (Figure 3), specificity was 0.98 (0.95–0.99) (Figure 3), PLR was 41.5 (18.1–95.2), NLR was 0.08 (0.03–0.19), DOR was 536 (128–2234). Figure 4 shows that there was heterogeneity among the studies. Figure 5 shows that the summary LRP and LRN for PCR was at the left upper quadrant (LUQ), revealing that PCR assay could be a good exclusion and confirmation standard for identifying ALK mutation. Summary receiver operator characteristic (SROC) curve was constructed based on the sensitivity and specificity of eligible studies, it’s corresponding area under the SROC curve (AUC) was 0.99 (0.98–1.00) (Figure 6), which suggested that PCR had a relatively high diagnostic performance in detecting ALK gene rearrangement of NSCLC patients. Fagan diagram (Figure 7) shows that post-test probability (91%) was larger than pre-test probability (20%) significantly, which meant the clinical utility of PCR in NSCLC patients for detecting ALK gene rearrangement was effective.

### Subgroup and meta-regression analysis

The pooled overall *I*^2^ values was 92.92%, *I*^2^ values for overall sensitivity and specificity were 85.43% and 93.80% respectively, which indicated that heterogeneity existing among the included studies. Figure 4 also suggested heterogeneity may exist across the eligible studies. Therefore, we conducted the meta-regression analysis to find out the source of heterogeneity with the type of specimen detected by PCR and FISH, the type of ALK gene fusion, the type of tissue used for detecting etc. Results were shown in Table 1, which indicated that language and the type of PCR were the sources of heterogeneity across the enrolled studies of sensitivity analysis and joint model analysis (Supplementary Figures 2 and 3).

**Table 1:** Results of meta-regression

Parameter	Sensitivity		Specificity		Joint model		
	**95% CI**	***p***	**95% CI**	***p***	**LRTChi2**	***P***	***I***^2^
Year of publication	0.96 (0.83–0.99)	0.58	0.97 (0.91–0.99)	0.48	4.12	0.13	51
Language	0.00 (0.00–1.00)	0.99	0.98 (0.83–1.00)	0.97	9.07	0.01	78
Country	0.89 (0.73–0.96)	0.57	0.99 (0.95–1.00)	0.62	4.24	0.12	53
Types of tumor	0.95 (0.73–0.99)	0.70	0.97 (0.86–0.99)	0.80	0.43	0.81	0
Types of tissue for detection	0.95 (0.81–0.99)	0.59	0.96 (0.88–0.99)	0.48	2.59	0.27	23
Types of ALK mutations	0.99 (0.91–1.00)	0.07	0.97 (0.77–1.00)	0.72	6.05	0.05	67
Types of material for FISH	0.90 (0.56–0.99)	0.81	0.94 (0.78–0.99)	0.26	1.83	0.40	0
Cells counted for FISH	0.92 (0.80–0.97)	0.88	0.98 (0.95–0.99)	0.99	0.25	0.88	0
Positive cutoff for FISH	0.85 (0.61–0.96)	0.34	0.99 (0.95–1.00)	0.05	4.36	0.11	54
FISH signal distance	0.92 (0.78–0.97)	0.89	0.97 (0.93–0.99)	0.72	0.61	0.74	0
Suppliers of FISH kit	0.86 (0.69–0.94)	0.37	0.97 (0.93–0.99)	0.69	3.79	0.15	47
Types of material for PCR	0.91 (0.73–0.97)	0.79	0.98 (0.93–0.99)	0.98	0.23	0.89	0
Types of PCR	0.20 (0.01–0.87)	0.03	0.69 (0.09–0.98)	0.06	6.74	0.03	70
Suppliers of ALK rearrangement Gene Diagnostic kit	0.81(0.40–0.96)	0.33	0.98 (0.91–11.00)	0.74	2.04	0.36	2
Suppliers of RNA extracted kit	0.90 (0.75–0.96)	0.64	0.98 (0.94–0.99)	0.98	1.23	0.54	0
Principle of PCR	0.98 (0.78–1.00)	0.37	0.97 (0.87–0.99)	0.78	2.00	0.37	0

FISH, fluorescence in situ hybridization; ALK, anaplastic lymphoma kinase; PCR, reverse transcriptase polymerase chain reaction.

## DISCUSSION

ALK is a member of receptor tyrosine kinase family and ALK translocations have been detected in various cancers, including NSCLC. With the development of targeted therapy, NSCLC patients with ALK fusions could benefit from ALK inhibitor. Therefore, it’s important to identify the ALK gene status to promote personalized cancer therapy. Even though the ALK fusion gene can be detected by various methods, such as FISH, IHC and RT-PCR, the best method to detect ALK alter remains controversial [33]. Therefore, we conducted the systematic review and meta-analysis to evaluate the diagnostic capacity of PCR.

According to the results of the meta-analysis, the overall pooled sensitivity was 0.92 (0.82–0.97), specificity was 0.98 (0.95–0.99), which suggested that PCR had a relatively high diagnostic accuracy in detecting ALK gene rearrangement of NSCLC patients. The DOR represents the value that combines sensitivity and specificity, ranging from 0 to infinity, with higher value meaning better discriminating ability [34]. We found the DOR was 536 (128∼2234), indicating that the discriminating ability of PCR was high. Additionally, the likelihood ratio (PLR and NLR) was used to estimate the diagnostic accuracy in clinical practice [35]. The PLR was 41.5 (18.1–95.2), which suggested that patients with cancer had about a 41.5 high chance of being PCR-positive compared to individual without ALK gene rearrangement. The NLR was 0.08(0.03–0.19), suggesting that the possibility of individuals with ALK gene rearrangement was 8% if the PCR test was negative. Since a PLR > 10 and NLR < 0.1 is the criteria of high accuracy, the results of PCR assay did achieve the requirement, indicating that PCR was enough for clinical confirmation and exclusion purpose. Figures 5 and 7 also showed the good confirmation and exclusion abilities for the clinical utility of PCR. An AUC of 1.0 represents the perfect discrimination ability, while an AUC closed to 0.5 indicates a poor test [36]. The pooled AUC in this study was 0.99 (0.98–1.00), which suggested the good discrimination ability for PCR.

Since the *I*^2^ of overall sensitivity and specificity were 85.43% and 93.80% respectively, indicating significant heterogeneity exiting across the included studies, therefore meta-regression analysis was performed to identify potential sources of heterogeneity. As a result, we found that language and the type of PCR were the reasons leading to heterogeneity among the enrolled studies of sensitivity analysis and joint model analysis. In the included studies, there were 4 studies published in Chinese language [20, 23, 24, 32], 17 studies published in English, 20 studies used the qRT-PCR and just 1 study used the end-point to detect ALK rearrangement [10]. As is shown in Figure 2, quality of articles in Chinese was lower than that published in English in this study. Additionally, qRT-PCR can detect the ALK rearrangement timely, thus end-point PCR can just detect ALK translocation at the end, which makes end-point PCR less sensitive than qRT-PCR. Though other factors such as FISH-positive cut-offs, the type of specimens for PCR, type of tissue used for detection and principle of PCR could affect the sensitivity or the specificity of FISH or PCR [12, 13, 30], but the result of the meta-regression failed to show that, which are probably associated with the limited studies and samples included.

Though our study was the first systematic review and meta-analysis to assess the diagnosis performances of PCR and FISH, there were still some limitations in our study. Firstly, we may miss some studies because of excluding abstracts of meetings and the ongoing studies, which may lead to the publication bias of this study. Secondly, whether the interpretation for the results of PCR and FISH was blind to the results of each other or not have not introduced clearly in many studies, which may impact on the methodological quality assessment of the included studies. Thirdly, the heterogeneity among the studies was significant, and the different language and different types of PCR were the source of heterogeneity.

In summary, our analysis showed that PCR has good discrimination ability to detect ALK rearrangement, but there still lack of high quality studies to verify the result. More studies especially large scale studies of high methodological quality are needed to verify the results of the study and to compare the diagnostic performances of PCR and FISH for detecting ALK rearrangement in NSCLC patients.

## MATERIALS AND METHODS

### Search strategy

We conducted a comprehensive literature search of PubMed, EMBASE, Web of Science, the Cochrane library, American Society of Clinical Oncology (ASCO), European Society for Medical Oncology (ESMO), China National Knowledge Infrastructure (CNKI), China Wan Fang databases and the Chinese biomedical literature database (CBM) to find relevant published articles on the diagnosis capacities of FISH and PCR for testing ALK rearrangement in NSCLC patients from inception to September 27, 2015 with the following terms without languages restricting: (ALK OR anaplastic lymphoma kinase) AND (NSCLC OR non-small cell lung carcinoma OR non-small cell lung cancer) AND (FISH OR fluorescent *in situ* hybridization) AND (PCR OR polymerase chain reaction). Additional articles were identified by screening the reference lists.

### Study inclusion and exclusion criteria

The titles, abstracts and full texts of the articles searched form the databases were screened by two researchers independently and the divergences were resolved by fully discussion or asking for help from a third researcher if the discussion failed to reach a consensus. Eligible studies had to meet the following criteria: (1) Patients in the studies were diagnosed as NSCLC; (2) Clinical studies evaluating PCR and FISH for the diagnosis of ALK mutations in NSCLC; (3) Studies provide sufficient data for constructing the diagnostic four fold (2×2) contingency table (i.e., true positive [TP], false positive [FP], false negative [FN], and true negative [TN]); (4) The article with the most details or the most recent was chosen if data or subsets of data were used in more than one articles. The exclusion criteria included: (1) Duplicate publication; (2) Reviews, case reports and letters to the editor, abstracts of meetings; (3) Unqualified or did not provide sufficient data.

### Data extraction and quality assessment

The following information was extracted from the enrolled studies: studies’ features (the last name of the first author, year of publication and country); participants’ general features (tumor type, number of samples); detection methods, type of specimen and data needed for analysis (TP, FP, FN and TN). We used the updated Quality Assessment of Diagnostic Accuracy Studies 2 (QUADAS-2) tool to evaluate the methodological quality of each study [37]. Meanwhile, the results of the quality assessment were presented by software RevMan 5.3.5.

### Statistical analysis

Software STATA 12.0 (Stata Corporation, College Station, TX, USA) was used to analyze the statistics. The pooled sensitivity, specificity, positive likelihood ratio (PLR), negative likelihood ratio (NLR), and diagnostic odds ratio (DOR) with their 95% confidence intervals (CIs) were calculated by the bivariate regression model [38]. Heterogeneity across the included studies was assessed by Cochran Q test and inconsistency index (*I*^2^), and *I*^2^ exceeds 50% indicates there is significant heterogeneity existing [39]. Meanwhile, the heterogeneity was also dissected by meta-regression analysis to explore potential causes. The summary receiver operator characteristic (SROC) curve was constructed based on the sensitivity and specificity of enrolled studies and we also calculated the corresponding area under the SROC curve (AUC) [40]. Additionally, we used the bivariate boxplot to assess the heterogeneity, evaluated the exclusion and confirmation capacities of the index test with the likelihood ratio scattergram, assessed the clinical utility of the PCR by the Fagan diagram.

### Role of the funding source

The funders had no role in study design, data collection and analysis, decision to publish, or writing of the manuscript.

## SUPPLEMENTARY MATERIALS FIGURES AND TABLE




