# Supplementary material for: Diagnostic accuracy of PCR for detecting ALK gene rearrangement in NSCLC patients: A systematic review and meta-analysis
Source: Oncotarget. 2017 May 17;8(43):75400–10. doi: 10.18632/oncotarget.17914 (PMC5650430; doi:10.18632/oncotarget.17914)
Supplement: Supplementary file 1 [file oncotarget-08-75400-s001.pdf]

Diagnostic accuracy of PCR for detecting ALK gene rearrangement in NSCLC patients: A systematic review and meta-analysis

SUPPLEMENTARY MATERIALS

Supplementary Table 1: Characteristics of studies included in the review. See Supplementary_Table_1

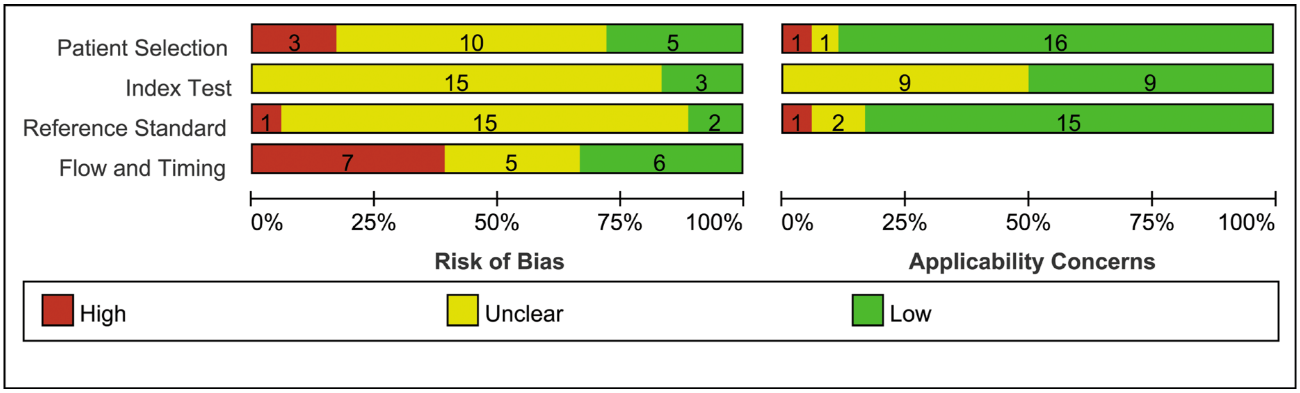

Supplementary Figure 1: Risk of bias and applicability concerns graph review authors' judgements about each domain presented as percentages across included studies.

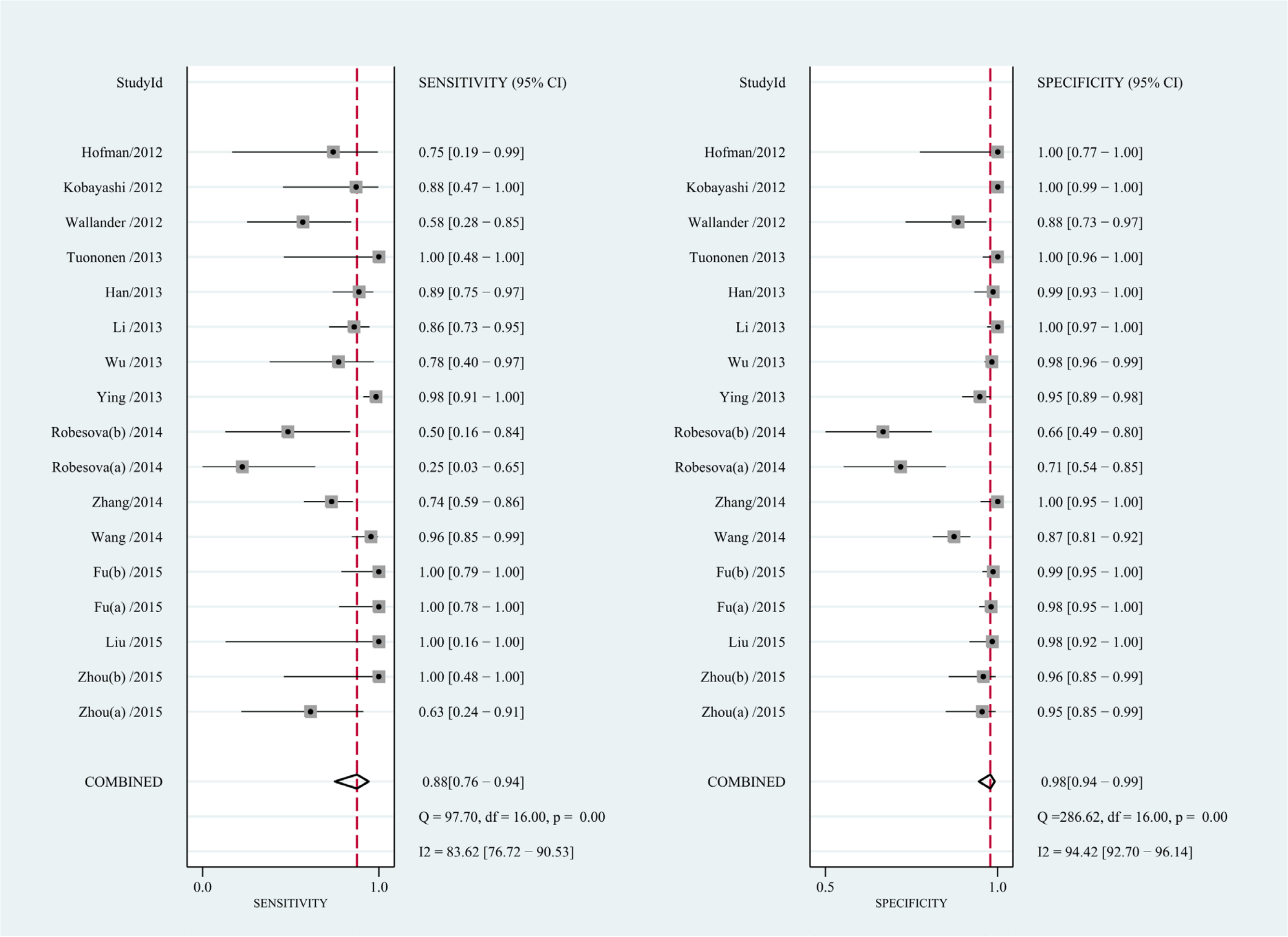

Supplementary Figure 2: Subgroup analysis in language of the included studies Subgroup analysis in language of the included studies.

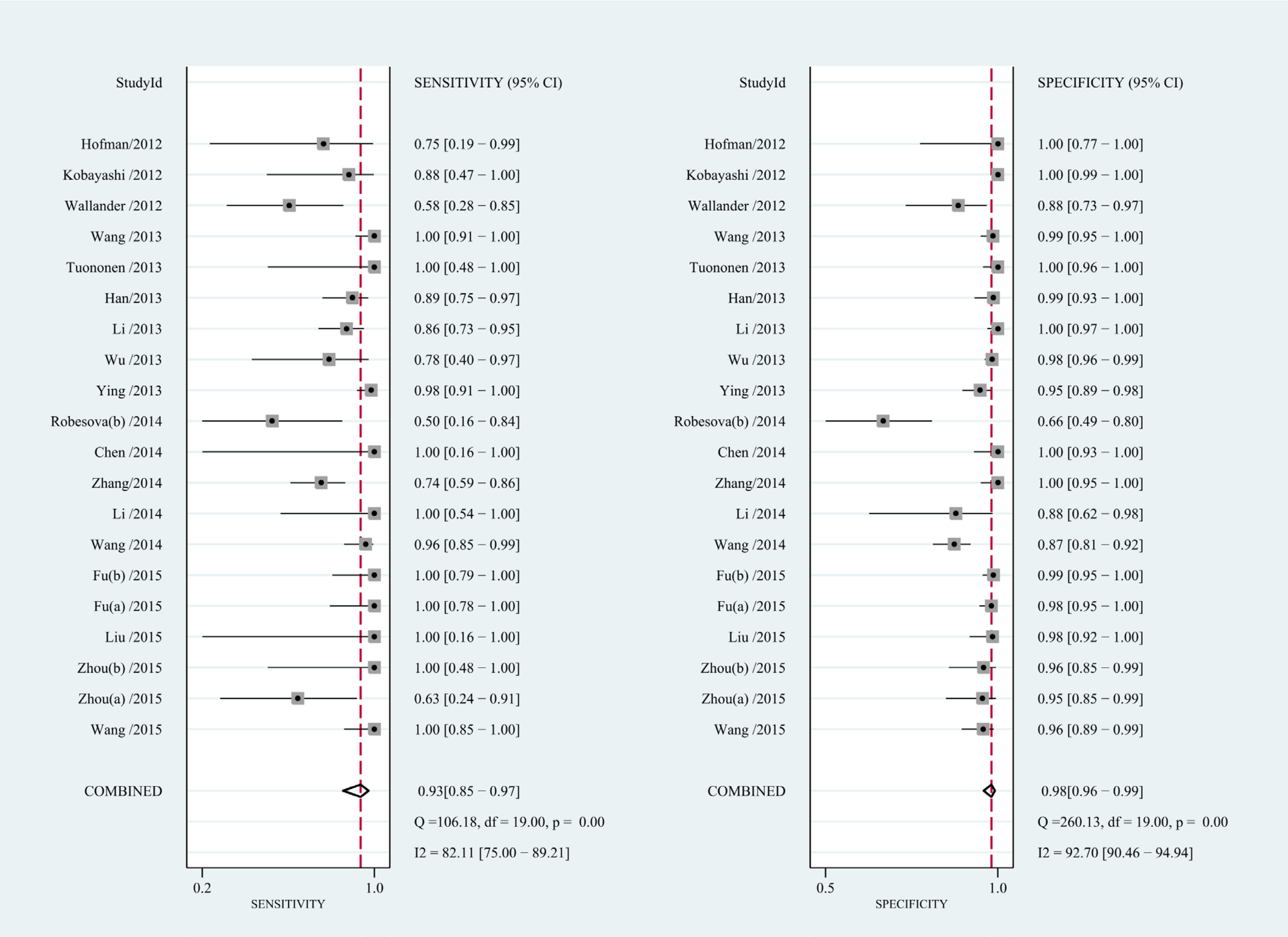

Supplementary Figure 3: Subgroup analysis in PCR of the included studies.
