# Supplementary material for: Diagnostic accuracy of PCR for detecting ALK gene rearrangement in NSCLC patients: A systematic review and meta-analysis
Source: Oncotarget. 2017 May 17;8(43):75400–10. doi: 10.18632/oncotarget.17914 (PMC5650430; doi:10.18632/oncotarget.17914)
Supplement: Supplementary file 2 [file oncotarget-08-75400-s002.docx]

| **Supplementary Table 1 Characteristics of studies included in the review** | | | | | | | | | | | | | | |
| --- | --- | --- | --- | --- | --- | --- | --- | --- | --- | --- | --- | --- | --- | --- |
| **Author** | **Study design** | **Country** | **Language** | **Tumor Type** | **Types of tissue for detection** | **Mutations** | **Types of material for FISH** | **Types of material for PCR** | **FISH** | **Types of PCR** | **TP** | **FP** | **FN** | **TN** |
| Wang 2015 | Diagnostic test | China | Chinese | NSCLC | tumor tissue | EML4-ALK | FFPE | FFPE | Cells counted: NR Cutoff of positive: NR Signal distance:NR Supplier: Abbott Molecular, Abbott Park, IL, USA | Types of PCR: qRT-PCR Principle of PCR: NR Suppliers of ALK fusion Diagnostic Kit : Amoy Diagnostics Company, China Suppliers of RNA extracted kit : Qiagen, Dusseldorf, Germany | 23 | 4 | 0 | 88 |
| Zhou(a) 2015 | Diagnostic test | China | English | Advanced NSCLC | tumor tissue | **EML4-ALK** | FFPE | FFPE | Cells counted: 50 Cutoff of positive: ≥15% Signal distance:≥2 Supplier: Abbott Molecular, Abbott Park, IL, USA | Types of PCR: qRT-PCR Principle of PCR: NR Suppliers of ALK fusion Diagnostic Kit : Amoy Diagnostics Company, China Suppliers of RNA extracted kit : Qiagen, Dusseldorf, Germany | 5 | 2 | 3 | 42 |
| Zhou(b) 2015 | Diagnostic test | China | English | Advanced NSCLC | pleural effusion | **EML4-ALK** | FFPE | FFPE | Cells counted: 50 Cutoff of positive: ≥15% Signal distance:≥2 Supplier: Abbott Molecular, Abbott Park, IL, USA | Types of PCR: qRT-PCR Principle of PCR: NR Suppliers of ALK fusion Diagnostic Kit : Amoy Diagnostics Company, China Suppliers of RNA extracted kit : Qiagen, Dusseldorf, Germany | 5 | 2 | 0 | 45 |
| Liu 2015 | Diagnostic test | China | English | NSCLC | pleural effusion | **EML4-ALK** | FFPE | FFPE | Cells counted: 100 Cutoff of positive: ≥15% Signal distance:＜2 Supplier: Medical Technologies , Inc., Beijing, China | Types of PCR: qRT-PCR Principle of PCR: NR Suppliers of ALK fusion Diagnostic Kit : Amoy Diagnostics Company, China Suppliers of RNA extracted kit : Qiagen, Hamburg, Germany | 2 | 1 | 0 | 63 |
| Fu(a) 2015 | Diagnostic test | China | English | Lung adenocarcinomas | tumor tissue | **ALK** | FFPE | FFPE | Cells counted: >50 Cutoff of positive: >15% Signal distance:>2 Supplier:Abbott Molecular, Des Plains, IL, USA | Types of PCR: qRT-PCR Principle of PCR: NR Suppliers of ALK fusion Diagnostic Kit : Self-designed Suppliers of RNA extracted kit : Qiagen, Hilden, Germany | 15 | 3 | 0 | 155 |
| Fu(b) 2015 | Diagnostic test | China | English | Lung adenocarcinomas | tumor tissue | **EML4-ALK** | FFPE | FFPE | Cells counted: >50 Cutoff of positive: >15% Signal distance:>2 Supplier: Abbott Molecular, Des Plains, IL, USA | Types of PCR: qRT-PCR Principle of PCR: TaqMan assays Suppliers of ALK fusion Diagnostic Kit : Self-designed Suppliers of RNA extracted kit : Qiagen, Hilden, Germany | 16 | 2 | 0 | 155 |
| Wang 2014 | Diagnostic test | China | English | Lung adenocarcinomas | NR | **ALK** | FFPE | FFPE | Cells counted: ≥100 Cutoff of positive: ≥15% Signal distance:NR Supplier: Abbott Molecular, Abbott Park, IL, USA | Types of PCR: qRT-PCR Principle of PCR: NR Suppliers of ALK fusion Diagnostic Kit : Amoy Diagnostics Company, China Suppliers of RNA extracted kit : NR | 44 | 20 | 2 | 134 |
| Li 2014 | Diagnostic test | China | Chinese | Lung adenocarcinomas | NR | **EML4-ALK** | FFPE | FFPE | Cells counted: ≥50 Cutoff of positive: ≥15% Signal distance:NR Supplier: GPMedical, Beijing, China | Types of PCR: qRT-PCR Principle of PCR: NR Suppliers of ALK fusion Diagnostic Kit : Amoy Diagnostics Company, China Suppliers of RNA extracted kit : Qiagen, Germany | 6 | 2 | 0 | 14 |
| Zhang 2014 | Diagnostic test | China | English | advanced nonsquamous NSCLC | tumor tissue | **EML4-ALK** | FFPE | FFPE | Cells counted: NR Cutoff of positive: NR Signal distance:NR Supplier: Abbott Molecular, Abbott Park, IL, USA | Types of PCR: qRT-PCR Principle of PCR: NR Suppliers of ALK fusion Diagnostic Kit : Amoy Diagnostics Company, China Suppliers of RNA extracted kit : Qiagen, Germany | 34 | 0 | 12 | 71 |
| Chen 2014 | Diagnostic test | China | Chinese | NSCLC | tumor tissue | **EML4-ALK** | FFPE | FFPE | Cells counted: NR Cutoff of positive: NR Signal distance:NR Supplier: Abbott Molecular, Abbott Park, IL, USA | Types of PCR: qRT-PCR Principle of PCR: NR Suppliers of ALK fusion Diagnostic Kit : Self-designed Suppliers of RNA extracted kit : Tian Gen Biotechnology Co., Ltd, Beijing, China | 2 | 0 | 0 | 50 |
| Robesova(a) 2014 | Diagnostic test | Czech republic | English | NSCLC | tumor tissue | **EML4-ALK** | FFPE and cytological smears | FFPE and cytological smears | Cells counted: 100 Cutoff of positive: >15% Signal distance:NR Supplier: ZytoVision, Bremerhaven, Germany | Types of PCR: End-point PCR Principle of PCR: NR Suppliers of ALK fusion Diagnostic Kit : Self-designed Suppliers of RNA extracted kit : Qiagen, Hilden, Germany | 2 | 11 | 6 | 27 |
| Robesova (b) 2014 | Diagnostic test | Czech republic | English | NSCLC | tumor tissue | **EML4-ALK** | FFPE and cytological smears | FFPE and cytological smears | Cells counted: 100 Cutoff of positive: >15% Signal distance:NR Supplier: Abbott Molecular, Abbott Park, IL, USA | Types of PCR: qRT-PCR Principle of PCR: TaqMan assay Suppliers of ALK fusion Diagnostic Kit : Self-designed Suppliers of RNA extracted kit :Qiagen, Hilden, Germany | 4 | 13 | 4 | 25 |
| Ying 2013 | Diagnostic test | China | English | Lung adenocarcinomas | tumor tissue | **EML4-ALK** | FFPE | FFPE | Cells counted: NR Cutoff of positive: NR Signal distance:NR Supplier: Abbott Molecular, Abbott Park, IL, USA | Types of PCR: qRT-PCR Principle of PCR: NR Suppliers of ALK fusion Diagnostic Kit : Amoy Diagnostics Company, China Suppliers of RNA extracted kit : NR | 62 | 7 | 1 | 126 |
| Wu 2013 | Diagnostic test | China | English | NSCLC | tumor tissue | EML4-ALK | FFPE | Fresh frozen | Cells counted: ≥100 Cutoff of positive: >15% Signal distance:≥2 Supplier:Abbott Molecular, Abbott Park, IL, USA | Types of PCR: qRT-PCR Principle of PCR: NR Suppliers of ALK fusion Diagnostic Kit : Self-designed Suppliers of RNA extracted kit : Invitrogen, Carlsbad, Calif | 7 | 5 | 2 | 291 |
| Li 2013 | Diagnostic test | China | English | Lung adenocarcinomas | tumor tissue | EML4-ALK | FFPE | Fresh frozen | Cells counted: 50 Cutoff of positive: >15% Signal distance:2 Supplier:Abbott Molecular, Abbott Park, IL, USA | Types of PCR: qRT-PCR Principle of PCR: NR Suppliers of ALK fusion Diagnostic Kit : Self-designed Suppliers of RNA extracted kit : Invitrongen Inc | 38 | 0 | 6 | 117 |
| Han 2013 | Diagnostic test | China | English | Lung adenocarcinomas and squamous cell carcinoma | NR | **EML4-ALK** | FFPE | FFPE | Cells counted: ≥50 Cutoff of positive: >15% Signal distance:>2 Supplier:Abbott Molecular, Abbott Park, IL, USA | Types of PCR: qRT-PCR Principle of PCR: NR Suppliers of ALK fusion Diagnostic Kit : Amoy Diagnostics Company,China Suppliers of RNA extracted kit :Qiagen, Hilden, Germany | 33 | 1 | 4 | 77 |
| Tuononen 2013 | Diagnostic test | Finland | English | NSCLC | tumor tissue | **EML4-ALK** | FFPE | FFPE | Cells counted: 50 Cutoff of positive: ≥15% Signal distance:≥2 Supplier:Abbott Molecular, Abbott Park, IL, USA | Types of PCR: qRT-PCR Principle of PCR: NR Suppliers of ALK fusion Diagnostic Kit : Amoy Diagnostics Company, China Suppliers of RNA extracted kit :Qiagen, Hilden, Germany | 5 | 0 | 0 | 82 |
| Wang 2013 | Diagnostic test | China | Chinese | NSCLC | tumor tissue | **ALK** | Fresh frozen and FFPE | Fresh frozen and FFPE | Cells counted: 100 Cutoff of positive: >15% Signal distance:>1 Supplier:Abbott Molecular, Abbott Park, IL, USA | Types of PCR: qRT-PCR Principle of PCR: SYBL Green Suppliers of ALK fusion Diagnostic Kit : Self-designed Suppliers of RNA extracted kit :Qiagen, Hilden, Germany | 38 | 2 | 0 | 137 |
| Wallander 2012 | Diagnostic test | America | English | Lung adenocarcinomas | tumor tissue | **EML4-ALK** | FFPE | FFPE | Cells counted: NR Cutoff of positive: ≥20% Signal distance:＜2 Supplier:Abbott Molecular, Abbott Park, IL, USA | Types of PCR: qRT-PCR Principle of PCR: NR Suppliers of ALK fusion Diagnostic Kit : Self-designed Suppliers of RNA extracted kit : Beckman Coulter Genomics, Danvers, Massachusetts | 7 | 4 | 5 | 30 |
| Kobayashi 2012 | Diagnostic test | Japan | English | NSCLC | tumor tissue | **EML4-ALK** | FFPE | FFPE | Cells counted: ≥200 Cells per 40 fields of tumor Cellss Cutoff of positive: obious documented case of a segregation probe Signal distance:NR Supplier: Self-designed | Types of PCR: qRT-PCR Principle of PCR: NR Suppliers of ALK fusion Diagnostic Kit : Self-designed Suppliers of RNA extracted kit :Qiagen, Hilden, Germany | 7 | 0 | 1 | 573 |
| Hofman 2012 | Diagnostic test | France | English | NSCLC | tumor tissue | **EML4-ALK** | FFPE | FFPE | Cells counted: 100 Cutoff of positive: ＞15% Signal distance:>2 Supplier:Abbott Molecular, Des Plaines, IL, USA | Types of PCR: qRT-PCR Principle of PCR: NR Suppliers of ALK fusion Diagnostic Kit : Self-designed Suppliers of RNA extracted kit :Qiagen, Hilden, Germany | 3 | 0 | 1 | 14 |
| NSCLC, non-small cell lung carcinoma; FISH, fluorescence in situ hybridization; ALK, anaplastic lymphoma kinase; RT-PCR, reverse transcriptase polymerase chain reaction; qRT-PCR, quantitative real time reverse transcription polymerase chain reaction; EML4-ALK, echinoderm microtubule-associated protein like 4-ALK; FFPE, formalin-fixed and paraffin-embedded; TP, true positive; FP, false positive; FN, false negative; TN, true negative; NR, not report. | | | | | | | | | | | | | | |
